# Characterization and Schwann Cell Seeding of up to 15.0 cm Long Spider Silk Nerve Conduits for Reconstruction of Peripheral Nerve Defects

**DOI:** 10.3390/jfb7040030

**Published:** 2016-11-30

**Authors:** Tim Kornfeld, Peter M. Vogt, Vesna Bucan, Claas-Tido Peck, Kerstin Reimers, Christine Radtke

**Affiliations:** 1 Department of Plastic, Aesthetic-, Hand- and Reconstructive Surgery, Hannover Medical School, Hannover 30625, Germany; Kornfeld.Tim@mh-hannover.de (T.K.); vogt.peter@mh-hannover.de (P.M.V.); bucan.vesna@mh-hannover.de (V.B.); Peck.Claas-Tido@mh-hannover.de (C.-T.P.); Reimers.kerstin@mh-hannover.de (K.R.); 2 Department of Plastic and Reconstructive Surgery, Medical University of Vienna, Währinger Gürtel 18-20, Vienna 1090, Austria

**Keywords:** spider silk, nerve graft, reconstruction, nerve surgery

## Abstract

Nerve reconstruction of extended nerve defect injuries still remains challenging with respect to therapeutic options. The gold standard in nerve surgery is the autologous nerve graft. Due to the limitation of adequate donor nerves, surgical alternatives are needed. Nerve grafts made out of either natural or artificial materials represent this alternative. Several biomaterials are being explored and preclinical and clinical applications are ongoing. Unfortunately, nerve conduits with successful enhancement of axonal regeneration for nerve defects measuring over 4.0 cm are sparse and no conduits are available for nerve defects extending to 10.0 cm. In this study, spider silk nerve conduits seeded with Schwann cells were investigated for in vitro regeneration on defects measuring 4.0 cm, 10.0 cm and 15.0 cm in length. Schwann cells (SCs) were isolated, cultured and purified. Cell purity was determined by immunofluorescence. Nerve grafts were constructed out of spider silk from *Nephila edulis* and decellularized ovine vessels. Finally, spider silk implants were seeded with purified Schwann cells. Cell attachment was observed within the first hour. After 7 and 21 days of culture, immunofluorescence for viability and determination of Schwann cell proliferation and migration throughout the conduits was performed. Analyses revealed that SCs maintained viable (>95%) throughout the conduits independent of construct length. SC proliferation on the spider silk was determined from day 7 to day 21 with a proliferation index of 49.42% arithmetically averaged over all conduits. This indicates that spider silk nerve conduits represent a favorable environment for SC attachment, proliferation and distribution over a distance of least 15.0 cm in vitro. Thus spider silk nerve implants are a highly adequate biomaterial for nerve reconstruction.

## 1. Introduction

More than 300,000 cases of peripheral nerve injuries are reported annually in the European Union [1]. Current gold standard in peripheral nerve surgery is the tension-free end-to-end suture of injured nerve tissue. In cases with a remarkable loss of nerve tissue and a resulting nerve gap, where a tension free end-to-end-suture is not achievable, an autologous nerve transplantation has to be performed. In general, autologous nerves for transplantation have to be surgically removed and then microsutured into the existing nerve defect [2]. As donor tissue, sensory nerves (e.g., sural nerves) are being used, resulting in a subsequent sensory loss at the donor site. An additional disadvantage is the inevitable limitation of donor tissue. Due to the limitation of donor nerves and the severe donor site morbidity with loss of sensitivity in the area of distribution, the development of surgical alternatives is desperately needed [3].

In cases with multiple nerve lesions the use of synthetic nerve grafts is indicated. Several materials have been approved for nerve surgery by the FDA (United States Food and Drug Administration) and are available for surgical treatment [4]. Current materials such as poly(DL-lactide-ε-caprolactone), polyglycic acid, chitosan and collagen are approved as nerve conduits for peripheral nerve surgery. Biodegradable poly(DL-lactide-ε-caprolactone) showed good regeneration on short nerve defects up to 2.0 cm in clinical multicenter studies [5]. Grafts produced out of polyglycic acid have been approved for surgical intervention by the FDA since 1999 and have occurred with full motor neuron recovery in a retrospective study on short gaps [6]. Another FDA-approved collagen-based nerve graft that is already commercially available for treating short nerve defects recently demonstrated neuroma formation after surgical treatment of a 2.0 cm nerve defect. This demonstrates that current collagen-based nerve grafts may not be adequate for a long nerve defect repair [7]. In general, commercial available nerve tubes are only approved for defect length up to 2.0 cm, thus emphasizing the need for development of scaffolds that could be used for large nerve defects [4]. 

Multichanneled grafts represent a new approach in the treatment of peripheral nerve defects. They are available in several different basic materials. Recently a collagen-based multichannel graft was tested on a 2.0 cm sciatic nerve defect model in rodent animals and achieved similar results compared to autologous controls [8]. Another approach is the use of chitosan. Chitosan is a polymer generated from chitin that is reported to enhance peripheral nerve regeneration for distances up to 1.5 cm. Compared to silicone and isografts, chitosan had similar or even superior regeneration potential in peripheral nerve defects [9,10]. Disadvantages include significant inflammatory processes during degradation in vivo [11].

Cadaveric decellularized allografts are a promising alternative to previously introduced artificial nerve grafts out of poly(DL-lactide-ε-caprolactone), polyglycic acid, chitosan and collagen as they are widely available and morphologically identical to autografts. Results of nerve regeneration with acellular pre-processed allografts were comparable to the current gold standard in nerve surgery [12].

Cadaveric allografts demonstrated superior regeneration of peripheral nerve defects compared to artificial silicone grafts. Moore et al. demonstrated a noticeable influence of pre-operative chemical treatment of nerve allografts to regeneration in vivo [13,14]. A major problem regarding allografts is the occurrence of inflammation and tissue rejection after implantation [15]. To avoid the reaction of the immune system, lifelong immunosuppression is be required. This often leads to chronic infections and malignant tumor formation from a long-term perspective [16,17].

However, tissue rejection is still observable, thus no complete immunosuppression is therapeutically indicated. On the other hand, improvement of nerve regeneration under tacrolimus was observed in experimental nerve reconstruction using allografts in rodent animals [18]. Thus, research for development of new procedure for nerve regeneration is from major interest, especially for reconstructive surgery.

To address these issues, an artificial biodegradable nerve graft constructed out of spider silk from *Nephila edulis* and a decellularized vessel was developed [19].

Spider silk is a unique material with remarkable mechanical properties, with tensile strength of 1.1 GPa and an elasticity of up to 35% by an average weight of 1.3 g/cm^3^. Thermal resistance is between −75 °C and 230 °C [20,21,22]. The main structure of major ampullate spider silk is characterized by a heavy chained fibroin with cumulated regions of glycine and alanine amino acid sequences that may contribute to these mechanical properties, as well as the structural and orientional order on the molecular level [23]. It is assumed that the secondary structure of β-sheet and the presence of proline contributes to this equally [24].

Silk constructs have already been successfully tested in nerve defect injuries as nerve implants in nerve regeneration [25], rodent fascia replacement [26]**,** and as a surgical suture material for microsurgical intervention [27]. No signs of tissue rejection and inflammation processes occurred [28,29].

In the current study, the experimental setting from Allmeling et al. [19] was modified to further improve spider silk nerve conduits. Highly purified Schwann cells were seeded on spider silk in the nerve construct to evaluate cell enhancement and proliferation of Schwann cells with different construct sizes (4.0 cm, 10.0 cm, and 15.0 cm). Additionally, cell adhesion, Schwann cell proliferation and migration throughout the spider silk nerve conduit was investigated in order to optimize the conduit for future consecutive in vivo experiments in long nerve gap injuries.

## 2. Results

**Figure 1:**
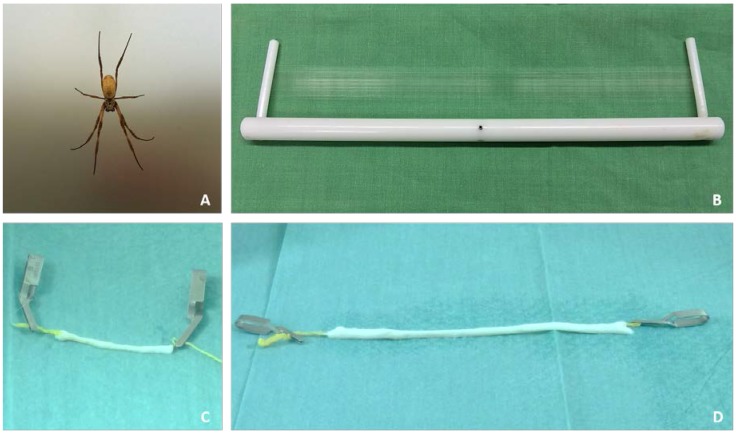
*Nephila edulis* and spider silk as an innovative biomaterial in nerve regeneration. (**A**) *Nephila edulis*; (**B**) Spider silk on 30.0 cm silk-collector; (**C**) 4.0 cm and (**D**) 15.0 cm spider silk nerve implant.

Spider silk was harvested from adult female *Nephila edulis* spiders (Figure 1A). The *Nephila edulis* spider is classified as a silk producing spider of family Nephilidae, of species *Nephila* which is endemic to eastern parts of Australia. Characteristic of the colloquially-named “golden orb weaver” is the individual pattern on the prosoma, the front body part of the spider [30]. With an average diameter of 1.5 cm–2.0 cm in the prosoma and the opisthosoma, *Nephila edulis* reach adulthood at the age of 5–6 months and are used for spider silk harvest thenceforward.

The harvested dragline silk is produced by the major ampullate and is naturally used by the spider itself as sort of safety leash to prevent falls from heights and for transverse pre-stressing of spider webs. These special drag line silk was applied as inner guidance structure in the described experiments. (Figure 1B).

Spider silk (as a natural biomaterial) can be harvested with the described method above at a rate of 75.0 mm per second from one spider with an estimated average diameter of 1.0–4.0 µm. Maximum harvest during one melting step of a single adult spider was 67,500 mm/15 min.

Ovine jugular veins were dissected from sheep. Veins were then decellularized over 14 days using lab standard protocol and prepared for cell culture. Veins were cut to required lengths of 4.0 cm, 10.0 cm and 15.0 cm. Then, 75× windings of spider silk were pulled through decellularized veins to form the inner lining of spider silk nerve implants. The constructed graft was watered using sterile phosphate-buffered saline (PBS) to avoid super contraction of spider silk during in vitro investigation. Figure 1C,D shows spider silk nerve implants of 4.0 cm and 15.0 cm prepared for Schwann cell pre-seeding. The spider silk is pulled through the vessel and fixed using sterile clips. Spider silk nerve grafts n = 2 were prepared for each time point with lengths of 4.0 cm, 10.0 cm and 15.0 cm.

**Figure 2:**
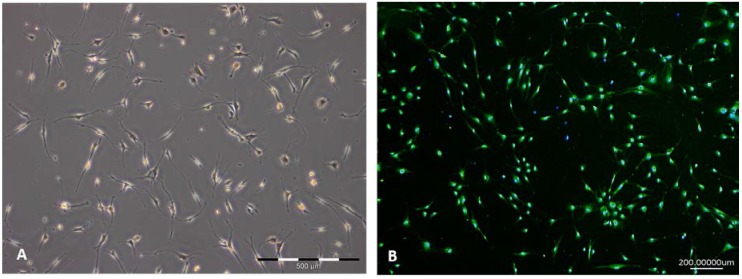
Schwann cell (SC) culture during purification process in vitro. (**A**) Schwann cells in vitro in a subconfluent culture; (**B**) Staining of Schwann cells with characteristic marker S100 (green), counterstained with 4′,6-diamidino-2-phenylindole (DAPI). The determined purity was 96.96%.

Schwann cells were isolated from sciatic nerves, cultured and purified by adherence. Cell viability was controlled using automated cell counting. The newly established culture protocol for Schwann cell isolation and subsequent culturing in vitro led to a cell purity of 96.96% after isolation (see below). The cell sizes ranged from 12.5 µm to 17.5 µm. Three percent dead cells were visible with an average cell size of 8.0 µm. Viability could be maintained during passaging. Figure 2A shows a characteristic Schwann cell culture during the purification process by adherence in passage one. Schwann cell-like cells with small flat bipolar extensions are visible. Slight contamination with bright flattened, triangular fibroblast-like cells (<3%) with multiple short cell extensions were seen.

Cells were stained with the characteristic Schwann cell marker Ca-binding protein (S100) to reveal Schwann cell purity. The purity of cultured cells was determined using S100 and 4′,6-diamidino-2-phenylindole (DAPI) counterstaining (Figure 2B) and revealed a total amount of 96.96% positively-labeled cells after two passages. S100 stained cells in green and DAPI cell nuclei were counterstained in blue. Single DAPI-positive stained nuclei unrelated to S100 positive perinuclear zones in green indicate contamination with different cell types.

Cells were detached, prepared as cell suspension with a concentration of 2 × 10^6^ per mL and 1000 µL and then injected with a Hamilton syringe into the described nerve conduits (Figure 1C,D). Each construct was seeded with 2 × 10^6^ cells. Cell attachment could be observed onto the silk within the first hour.

Spider silk nerve grafts of different sizes (4.0 cm, 10.0 cm and 15.0 cm), pre-seeded with Schwann cells were observed in vitro for 7 and 21 days in order to evaluate cell viability.

**Figure 3:**
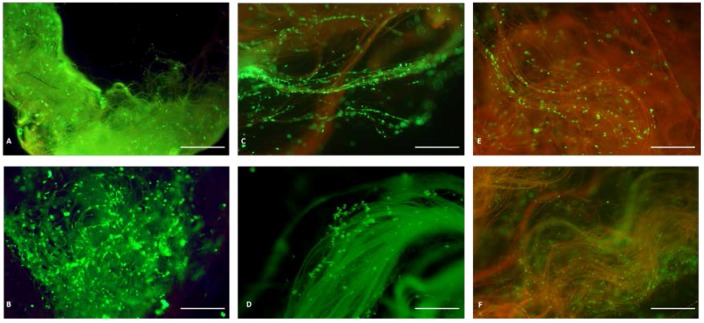
Viability assay of spider silk nerve construct seeded with Schwann cells and cultured after 7 and 21 days of observation. Pre-settled spider silk nerve conduits were cultured for 7 and 21 days. Green cells are vital. Spider silk autofluorescence are shown orange-red. The number of cells increased by proliferation from day 7 to day 21 on a 4.0 cm construct (**A**,**B**). Diffuse population of Schwann cells on a 10.0 cm construct (**C**) increased during observation time of 21 days (**D**). On the 15.0 cm construct, at 7 days only a few SCs could be observed (**E**) but upon intense proliferation, the spider silk construct was fully populated with SC after 21 days (**F**). Cells are highly vital (green), indicative of sufficient nutritive substances in the central segments of the 15.0 cm spider silk nerve conduit. Scale bars in A–F pertain to 500 µm.

Further characterization of the spider silk/vein conduit was performed after 7 days and 21 days of co-culturing. Viability controls were performed on each conduit using live/dead staining (Figure 3) at individual endpoints. Ratio between vital and non-vital cells was raised to quantify viability index, independent of construct length. Red staining indicates dead cells and green staining of cells is indicative of vital metabolic active cells. Moreover, the assay revealed that cells were maintained viable in a 3D structure within the conduit. No signs of extensive cell dead or cell lysis were visible throughout the experimental observation period. Spider silk appears with slight auto fluorescence as previously described [19]. Figure 3A–E shows lightly green-yellow to orange-red autofluorescence of natural untreated silk after cell culture.

In general, spider silk nerve constructs independently from vessel length were fully filled throughout the construct with Schwann cells in the inner central segment after 21 days of co-culturing. It is important to note that parallel aligned spider silk fibers in the center of the spider silk construct were excessively covered with multiple layers of Schwann cells.

### 2.1. The 4.0 cm Spider Silk Nerve Conduit

The 4.0 cm spider silk nerve conduit was entirely populated with cells at day 7. Schwann cells attached directly onto the spider silk fibers. No direct cell-to-cell contacts were evident. Bidirectional longitudinal growth from both ends to the central segments occurred. The majority of silk fibers were covered with longitudinally aligned Schwann cells. Spider silk can be seen by its slight green autofluorescence in addition to the live/dead staining of the SCs. At day 7, the vast majority of cells attached to the silk were alive as demonstrated by the live/dead staining. No obvious dead cells could be observed at this point. A confluency of 60% was determined after one week in vitro within the spider silk construct (Figure 3A).

At 21 days of cell culture, the number of viable cells increased by ~51% on the 4.0 cm graft. Multilayered cell growth on spider silk could be observed. Homogenous cell growth of Schwann cells with continuous distribution through artificial nerve graft was visible (Figure 3B). Nearly all silk fibers were covered throughout the whole 4.0 cm conduit after three weeks. All cells appear vital i.e., there was no evidence for dead cells with a cell viability after day 21 >99% with a confluency of 80%.

### 2.2. The 10.0 cm Spider Silk Nerve Conduit

The 10.0 cm nerve grafts seeded with Schwann cells showed longitudinal multilayer cell coverage of single fibers after one week of cultivation. Cells attached to the silk fibers and started to proliferate through the inner layer of the conduits on selected individual fibers (Figure 3C). Cells accumulated on fibers in relation to the center of the cylindrical conduit with accumulation of viable cells up to both ends of the conduit. Cell number decreased to inner segments of 10.0 cm graft but revealed still significant SC numbers. However, a confluency of ~25% was determined after day 7 with a cell viability of >99%.

After three weeks of culture, the 10.0 cm construct reached a confluency of ~70%. Cell proliferation set up a three-dimensional network (Figure 3D) within the nerve graft. Cell number increased by ~31.25% during observation time. Cell growth on single fibers was still ostentatious. Few fibers with relation to conduits wall with considerably lower cell numbers. There were no signs of significant numbers of dead cells in the entire length of the conduit after 21 days.

### 2.3. The 15.0 cm Spider Silk Nerve Conduit

Most fibers in the central segment of the spider silk fiber bundle of the inner layer were covered with cells (Figure 3E). Peripheral fibers were without or had significantly lower cell numbers (Figure 3E). Cells occurred in small groups, indicative of necessary cell-to-cell contact for proliferation with noticeable gaps between the fibers. Cell number was reduced in the inner segments of 15.0 cm conduit. Decisive confluency of ~10% was compiled. Life/dead staining was performed with ~90% green vital cells. The majority of Schwann cells remain vital with no difference regarding cell viability from peripheral to central segments. A small number of dead cells were visible on peripheral silk fibers.

At 21 days of SC culture within the spider silk conduit, cell numbers were further increased by 66%. A confluency of ~80% was reached. Silk fibers were surrounded by longitudinally aligned SCs along the spider silk. Figure 3F shows spider silk from the inner segment of the 15.0 cm spider silk nerve conduit after 21 days. Only a few dead cells labeled in red were visible whereas high numbers of viable cells (green) were present. Here, we could demonstrate that the silk provided an optimal guidance structure for up to at least 15.0 cm within the conduit in vitro, on which Schwann cells were equally distributed and attached and on which high cell viability (>95%) could be maintained in long-term culture.

## 3. Discussion

Injuries of the peripheral nerves are still a challenging problem in plastic and reconstructive surgery. After severe injury of a peripheral nerve by traumatic transection or nerve crush by pressure, Wallerian degeneration provides an environment for nerve regeneration by removal of degenerating peripheral nerve and reassembling of peripheral Schwann cells. Within 24–48 h, Wallerian degeneration is induced. One week post-injury, the Schwann cells in the distal stump are stimulated by neuroregulin I to induce proliferation and enforce production of myelin followed by basal lamina tubes (bands of Bünger) [31,32], indicating the crucial and essential role of Schwann cells in this regeneration process. Therefore, the combination of a favorable biomaterial for enhancement of nerve regeneration in combination with Schwann cells is a logical step.

Spider silk as a biomaterial has already shown its unique properties with regard to nerve regeneration and cell enhancement in vitro and in vivo. Recently, it was reported that spider silk served as guidance material for human model neurons in vitro and that spider silk enhanced the development of ganglion like structures within one month [33]. Previously it was shown that NIH/3T3-fibroblasts can grow on spider silk and lead to a dense network on the woven spider silk frames [34]. Moreover, it was demonstrated that spider silk can promote axonal regeneration in long, gaped peripheral nerve defects over a distance of 6.0 cm in sheep sciatic nerves. After 10 months the nerve defect was histologically and functionally regenerated and the spider silk completely degraded [25].

The results of the present study are very promising with regard to nerve regeneration on nerve defects over a distance of 6.0 cm. Cell growth in artificial nerve grafts of up to 15.0 cm is possible and not limited due to an insufficient distribution of nutritive substances. In particular, the combination of silk and Schwann cells seems to be advantageous. Due to this major task in nerve regeneration, the current opinion is that Schwann cells enhance and initiate the regeneration of the peripheral nerve [35]. One approach in surgical nerve repair is the use of artificial nerve grafts pre- or intraoperative settled with autologous Schwann cells [36,37]. Hadlock et al. introduced a new artificial nerve graft with rolled monolayers of Schwann cells. They were able to show that recovery in cell-free grafts are inferior to grafts seeded with Schwann cells or autologous nerve grafts [38]. Hoben et al. compared acellular allografts and allografts pre-seeded with Schwann cells and acellular allografts combined with vascular endothelial growth factor (VEGF) with regard to axon regeneration in a 2.0 cm sciatic nerve injury model in rodents. The results demonstrated clearly superior regeneration in Schwann cell pre-seeded allografts compared to acellular allografts alone, which was inferior to the current gold standard of autologous nerve grafts [39].

From this point of view, Schwann cell survival in synthetic nerve graft is the key for successful peripheral nerve regeneration. In this study, we examined the Schwann cell survival in an artificial nerve graft filled with spider silk in three different construct sizes (4.0 cm, 10.0 cm and 15.0 cm) for nerve gap implantation. As there is no experience with cell growth and cell survival with respect to long nerve defects >6.0 cm, it was necessary to initially investigate cell viability, attachment and growth in the described spider silk nerve conduits >6.0 cm in vitro before application in vivo. Therefore, an in vitro testing for spider silk nerve grafts up to 15.0 cm implant length was performed.

Schwann cell growth on spider silk was investigated by Allmeling et al. in 2006 [19]. Schwann cells were seeded on spider silk fibers with an average length of 5.0 cm during an in vitro experiment. Cell adherence to the spider silk and viability was controlled after 15 min, 24 h and 48 h. Here, long-term characterization was not explored, which is inevitable and crucial for nerve regeneration. Moreover, the effect in the complex structure of a channeled conduit was not explored. To our knowledge, no sufficient studies about long term culture of Schwann cells on nerve conduits over 5.0 cm have been performed. In the present study, Schwann cell adhesion, viability, adhesion and proliferation over a relatively long-term observation period in a 3D-structure was determined at different time points. These are important data and an inevitable prerequisite for further in vivo applications in order to enhance long nerve gap injuries extending 6.0 cm. 

Several protocols describe various methods for Schwann cell purification. Cell purity is essential given that contaminating cells such as fibroblasts have a higher proliferation index and would overgrow the Schwann cells in short periods. Moreover, many protocols result in a certain amount of Schwann cell loss during the purification process [40]. Schwann cell enrichment with selection techniques e.g., magnetic beads, can influence typical Schwann cell characteristics upon further cell cultivation. The most used protocols described in the literature are based on purification steps by adherence in combination with different medium supplements or the purification by magnetic/immunologic labeling [41]. A major advantage of magnetic/immunologic cell sorting techniques is the speed of purification. While one can reach highly purified cells within 5–9 days [42] by magnetic cell sorting, at least 21 days are needed for traditionally-used by adherence methods [43]. In this study, the protocol from Niapour et al. was slightly modified by using Melanocyte Basal Medium + Melanocyte Growth Medium Supplement Mix [43] to further optimize results. Thus, a highly purified culture of Schwann cells was reached. A new aspect is that the medium-based purification process only takes about seven days and cells are less senescent.

Recently, spider silk was demonstrated to be not only a cell carrier but also to act as a drug carrier. Brooks et al. reported that spider silk and silkworm silk are suitable for mucosal medical drug delivery due to their uniquely viscous properties [44]. Pritchard et al. described spider silk polymers suitable for long term drug application by using silk as a drug depot that slowly degrades and releases drugs after intravenous application or as a drug implant [45]. 

Thus, one could imagine further-improved nerve conduits based on silk with, e.g., binding of neuron-specific protein gene product 9.5 (PGP 9.5) protein onto the silk to enhance Schwann cell migration and proliferation or loading the silk with chondroitinase ABC (ChABC) or glial cell-derived neurotrophic factor (GDNF) to improve axonal regeneration. In other studies, it was demonstrated that nerve growth factor (NGF)-loaded nerve conduits provide similar results to autogenous nerves, and can promote axonal regeneration, improve the myelination and accelerate the functional nerve regeneration [46,47].

In summary, the present study revealed that Schwann cell proliferation throughout spider silk nerve implants of various sizes (4.0 cm, 10.0 cm and 15.0 cm) is possible. Live/dead staining demonstrated that SCs maintained viable during observation time of 21 days and appeared with an arithmetically averaged proliferation index of at least 49.42%. The number of vital cells increased within 21 days on the 4.0 cm spider silk implant, by ~51%. The light population of Schwann cells on the 10.0 cm construct built up a three-dimensional network during an observation time of 21 days with a cell proliferation index of ~31.25%. There was a small population of Schwann cells on the 15.0 cm construct after 7 days, however, the cell number increased about ~66% over three weeks. This indicates that the spider silk nerve implants are an adequate environment for Schwann cell distribution, proliferation and migration for distances up to 15.0 cm. 

## 4. Materials and Methods

### 4.1. Harvest of Spider Silk

Natural spider silk from the major ampullate of the female spiders of species *Nephila edulis* was used. The animals were kept in the lab´s own facilities at 29 °C room temperature and a humidity between 70% and 85%. Spiders were watered daily and nourished with *Acheta domesticus* twice a week and after each melting/harvesting step. Spider silk was harvested from spiders twice a week. Spider silk was woven on 30.0 cm polytetrafluorethylene frames with a maximal winding number of 225 (Figure 1B). Through an automatic lateral rotation of the motor assisted spinning machine, spider silk fibers were aligned in parallel with an average interval of <1 mm. (Figure 1B) and used for inner lining of the decellularized vessels. During each harvest, approximately 67.5 m of spider silk fibers were gained. Only dragline silk was used for this study. The silk was then sterilized and stored for further usage. 

### 4.2. Schwann Cell Isolation from Rodents

Adult male Wistar rats (weight 250–300 g) were anesthetized and sacrificed according to the German animal welfare law. Sciatic nerves were removed. Isolation followed the protocol of Radtke et al. 2005 [48].Cell growth in culture was controlled daily by light microscopy.

### 4.3. Purification of Schwann Cells by Adherence

After three weeks, 6-well- dishes were confluent. Cultured Schwann cells were transferred to a T-75 poly-l-lysin (PLL)-coated flask (TPP, Biochrome, Berlin, Germany). Then cells were detached using 0%, 25% Trypsin/EDTA (Biochrome, Germany). Cells were re-suspended in Melanocyte Growth Medium (PromoCell, Germany) and 1% Penicilin/Streptomycin +0.7 µL Forskolin (Sigma Aldrich, St. Louis, MO, USA)/mL Medium and transferred to a PLL-coated T-75 Flask. Change of medium was performed once per week. Progress in purification was observed daily in light microscopy.

### 4.4. Preparation and Decellularization of Vessels

Vessels were dissected from goats from experiments with different purposes. Vessels were decellularized following lab standard protocols.

### 4.5. Artificial Nerve Grafts

Decellularized vessels were cut to the required length (4.0 cm, 10.0 cm and 15.0 cm). 75× windings of spider silk fibers were pulled through vessels. Silk was fixed with sterile microsurgical serrefines at the proximal and distal ends (see Figure 1C,D). All steps were performed under sterile conditions. Twelve spider silk nerve implants were prepared for in vitro studies. There were two implants of 4.0 cm, 10.0 cm and 15.0 cm per time point, respectively. 

### 4.6. Schwann Cell Pre-Seeding of Spider Silk Nerve Implants

Cells were detached, prepared as a cell suspension with a concentration of 2 × 10^6^ per mL and 1000 µL, and then injected with a Hamilton syringe into the described nerve conduits (Figure 1C,D). Each construct was seeded with 2 × 10^6^ cells. Cell attachment on spider silk was controlled by light microscopy on peripheral zones between vessel and silk.

### 4.7. Cell Counting

Cell counting was either performed using automated counting from Software Image J [49] or by manual counting after analyzing cell morphology and counterstaining S100/DAPI. Prior to cell counting within the spider silk nerve implant, vessels were removed. Only cells attached to spider silk were manually counted. Total cell numbers in image sections were counted and sorted by color. Percentage of red and green cells were determined. To evaluate a proliferation index, total cell numbers in regard to endpoints were determined and set into relation respectively. Confluency within the spider silk nerve implants was determined.

### 4.8. Immunofluorescence

Cells were stained rabbit anti rat S100 (rabbit anti rat; DAKO Agilent Technologies, Santa Clara, CA, USA). Cells were seeded in the first passage on 6-well object slides coated with poly-l-lysin (PLL) and stained according to the following protocol. Cells were fixed with 4% paraformaldehyde (20 min). Blocking was conduct with 2% fetal calf serum (FCS)/PBS. Incubation with primary antibody S100 (rabbit anti-rat; DAKO, Agilent Technologies, Santa Clara, CA, USA) was performed at a 1/400 dilution in 1% FCS in PBS overnight at 4 °C in a humidified chamber. For visualization, the secondary antibody Alexa Fluor 488 (goat anti-rabbit IgG; chicken anti-rabbit, 495–519 nm, green fluorescent; Invitrogen, Carlsbad, CA, USA) was used. Slices were examined and cells quantified with a fluorescence microscope (Zeiss).

### 4.9. Live Dead

To control enhancement, viability and populated cell growth on the constructs, live/dead staining was performed. Construct was washed with PBS w/o. Subsequently cells were stained with 5 µL calcine-acetoxymethyl (495/517 nm, green, live cells; Life Technologies, Carlsbad, CA, USA) and 20 µL Ethidium Homodimere-1 (528/617 nm, red, dead cells; Life Technologies, Carlsbad, CA, USA), dissolved in 10 mL PBS w/o. Olympus Fluorescent microscope was used to analyze the grafts. 

## 5. Conclusions

Spider silk nerve conduits for peripheral nerve repair after nerve gap injury represent a favorable environment for SC attachment, proliferation and distribution over a distance of least 15.0 cm in vitro, providing optimal conditions for artificial nerve conduits based on spider silk as a highly biocompatible material for subsequent nerve growth enhancement in vivo. Here, it was demonstrated that cell attachment with high viability of Schwann cells and proliferation in vitro under standardized conditions in artificial nerve grafts filled with spider silk is possible, at least up to a construct length of 15.0 cm. These results are very promising for extended nerve defect reconstruction by spider silk nerve constructs with regard to future in vivo applications.

## Abbreviations

ChABC	Chondroitinase ABC
DAPI	4′,6-diamidino-2-phenylindole
DOAJ	Directory of open access journals
EDTA	Ethylenediaminetetraacetic acid
FCS	Fetal calf serum
GDNF	Glial cell-derived neurotrophic factor
LD	linear dichroism
MDPI	Multidisciplinary Digital Publishing Institute
NGF	Nerve Growth Factor
PBS	Phosphate Buffered Saline
Pen/Strep	Penicillin/ Streptomycin 1:1
PGP 9.5	Protein gene product 9.5
PLL	Poly-l-Lysine
SC	Schwann cell
S100	Ca-binding Protein
TLA	Three letter acronym
VEGF	Vascular Endothelial Growth Factor
